# Real-world Canadian data on belumosudil therapy in heavily pretreated patients with steroid-refractory chronic graft-versus-host disease: treatment outcomes and risk factor analysis for failure-free survival

**DOI:** 10.1007/s00277-025-06661-y

**Published:** 2025-10-25

**Authors:** Sergio Rodriguez-Rodriguez, Nihar Desai, Christopher Lemieux, Keven Vachon, Kareem Jamani, Mohamed Elemary, Tommy Alfaro-Moya, Eshrak Al-Shaibani, Ivan Pasic, Igor Novitzky-Basso, Fotios Michelis, Auro Viswabandya, Rajat Kumar, Jonas Mattsson, Arjun Law, Sylvie Lachance, Dennis Dong Hwan Kim

**Affiliations:** 1 Present Address: Princess Margaret Cancer Centre, Faculty of Medicine, University Health Network, University of Toronto, 610 University Ave. OPG Rm 6-222, Toronto, ON M5G2M9 Canada; 2 Centre hospitalaire universitaire (CHU) de Québec, Université Laval, Québec, Québec Canada; 3 Arthur Child Comprehensive Cancer Centre, University of Calgary, Calgary, AB Canada; 4 Saskatoon Cancer Agency, University of Saskatchewan, Saskatchewan, SK Canada; 5 Institut d’Hématologie, Oncologie, Greffe et Thérapie Cellulaire, Hôpital Maisonneuve Rosemont, Department of Medicine, Université de Montréal, Montréal, Canada

**Keywords:** Chronic graft-versus-host disease, Belumosudil, Steroid-refractory chronic GvHD, Hematopoietic stem cell transplantation, Real-world experience, Failure-free survival.

## Abstract

Chronic graft-versus-host disease (cGvHD) remains one of the common causes of morbidity and mortality after allogeneic hematopoietic stem cell transplantation. Belumosudil (BEL), a selective ROCK2 inhibitor, has immunomodulatory and anti-fibrotic properties, offering a new therapeutic option. Real-world data (RWD) in heavily pretreated patients remain limited, particularly for combination of BEL with ruxolitinib (RUX). We conducted a multicenter, real-world study in 46 patients treated for refractory cGvHD with BEL under a Canadian compassionate program. Treatment outcomes were assessed using the NIH consensus response criteria for overall response rate (ORR), failure-free (FFS), overall survival (OS), and safety. Forty-six patients were included with a median follow-up of 11.4 months; the best ORR was 52% (*n* = 20/38). The FFS and OS rates at 12 months were 64.3% and 91.1%, respectively. Steroids were discontinued in 73% at 12 months. BEL combination therapy with RUX exhibited equivalent treatment outcomes to BEL monotherapy, although patients treated with drug combination presented with more advanced form of GvHD and mostly failed RUX therapy. A prognostic risk model based on prior acute GvHD and involvement of ≥ 4 organs effectively stratified FFS at 12 months: 100% with no risk factors, 75.8% with one, and 30% with two risk factors (HR 3.91, 95% CI 1.58–9.67, *p* = 0.003). BEL demonstrated durable efficacy and acceptable safety in heavily pretreated cGvHD. BEL treatment was associated with a high probability of corticosteroid withdrawal. Risk stratification by disease burden and prior aGvHD identified distinct prognostic groups, informing patient selection and future therapeutic strategies.

## Introduction

Despite the development of new effective modalities for its prophylaxis, chronic graft-versus-host disease (cGvHD) remains a significant challenge in the management of survivors following allogeneic hematopoietic stem cell transplantation (HCT). It is a heavy burden contributing to the morbidity and mortality of a significant number of patients. While the introduction of newer agents such as ruxolitinib (RUX) or ibrutinib (IBR) has expanded the therapeutic landscape of cGvHD treatment, they have shaped our current practice but have had little to no impact on patients with irreversible fibrotic and sclerotic forms, a continuing therapeutic challenge [1]. These forms of cGvHD are often resistant to conventional treatments, leaving limited options for effective management [1, 2]. 

Belumosudil (BEL) is a selective Rho-associated coiled-coil kinase 2 (ROCK2) inhibitor with immunomodulatory effects in preclinical and early clinical studies [3, 4]. It has been shown that BEL reduces cytokine production and decreases STAT3, inhibiting Th17 cells while augmenting STAT5, boosting regulatory T-cells [3, 5, 6]. It decreases collagen and extracellular matrix production, targeting key mechanisms involved in the pathogenesis of fibrosis-related cGvHD [7, 8]. Its efficacy, particularly in managing the fibrotic component of cGvHD, constitutes an area of great interest.

In the ROCKstar study, patients received BEL either 200 mg daily or twice daily if on proton pump inhibitor, achieved a best overall response rate (ORR) of 74% and 77%, respectively [3]. When analyzing organ-specific responses, the best ORR was reported at 71% in the joints/fascia, 69% in the lower gastrointestinal tract, 55% in the mouth, 37% in the skin, and 26% in the lungs, respectively. The CR rates for the same organs were 20%, 61%, 44%, 16%, and 13%. Similar results were found in an open-label study with BEL 200 mg daily as a second or subsequent line of treatment, with a best ORR of 80% in the joints/fascia, 67% in the mouth, 20% in the eyes, and 54% in the skin [4].

While early clinical studies reported that BEL improved overall responses and suggested its capacity to reverse fibrosis [7], it is of interest to confirm these results in current clinical practice given the difficulties to apply the same stringent inclusion/exclusion criteria and replicate the results of clinical trials in day-to-day practice. The ROCKstar study excluded patients with liver enzyme abnormalities with transaminase levels above 5 times the normal value [3], whereas in real-world data (RWD), BEL is being used to treat hepatic GvHD in more heavily pretreated patients. Also, there are relatively few RWD studies on the efficacy of BEL in heavily pretreated and RUX refractory patients [9–11].

Many previous studies have evaluated combination of BEL with other treatment modality for cGvHD treatment for its feasibility and therapeutic efficacy. One option is combining BEL with RUX, aiming to enhance therapeutic synergism between the antifibrotic property of BEL and the strong anti-inflammatory activity of RUX [12]. Such a combination has been reported feasible and safe, lacking drug-drug interactions, adverse drug reactions, or deleterious infections rates [9, 13].

This study aimed to report RWD in patients treated with BEL as monotherapy or as combination treatment with RUX through the Canadian compassionate use program, which became available in March 2023 after Health Canada approval. We assessed its effectiveness, focusing on ORRs based on the National Institutes of Health (NIH) consensus response criteria, failure-free survival (FFS), and analyzed risk factors for FFS in patients with chronic cGvHD treated with BEL following the failure of multiple lines of therapy.

## Methods

### Patient characteristics

This retrospective, multicenter study aims to evaluate the efficacy, FFS, and overall survival (OS) in patients treated with BEL for corticorefractory or dependent cGvHD. We also aim to identify risk factors for FFS following BEL therapy in cGvHD patients who failed multiple lines of treatment. Data were collected from medical records of 46 patients treated under the compassionate use program for cGvHD at five Canadian centers: Princess Margaret Cancer Centre (Toronto; *n* = 26), Université de Montréal (Montreal; *n* = 10), University of Calgary (Calgary; *n* = 4), Université Laval (Quebec City; *n* = 3), and University of Saskatchewan (Saskatoon; *n* = 3), enrolled between March 2023 and June 2024. All research was conducted according to the Declaration of Helsinki. The Research Ethics Board approved the study at the University Health Network, Toronto (REB #19–5354), and each institution.

### Retrospective data collection

Baseline characteristics, prior cGvHD treatments, and details of BEL administration were captured through medical chart review, distinguishing between BEL monotherapy and combination treatment. Objective response to BEL was assessed according to the NIH consensus response criteria as a standard of care and captured retrospectively through the electronic medical record review. Adverse events (AEs) were classified according to the Common Terminology Criteria for AEs from the Cancer Therapy Evaluation Program.

### Definition of treatment outcomes and statistical endpoints

To evaluate BEL treatment outcomes, multiple parameters were analyzed, including the ORRs at 3, 6, and 12 months, the best ORR, steroid dose reduction, failure rate to BEL treatment, the FFS and OS at 12 months, and the photographic range of motion (P-ROM) improvement.

The ORR was defined as the proportion of patients with a complete (CR) or a partial response (PR); the best ORR was considered an ORR at any time during treatment. For steroid dose reduction, longitudinal changes were assessed and calculated based on the patient’s daily dose per body weight (mg/kg/day) before BEL therapy and at 3, 6, and 12 months following BEL treatment. We analyzed the proportion of patients on steroid doses ≤ 0.5, 0.2, and 0.1 mg/kg/day, and successful discontinuation longitudinally at 0, 3, 6, and 12 months. The cumulative incidence of steroid discontinuation was calculated from BEL start to complete discontinuation of steroid, while considering BEL discontinuation and death as competing events.

Failure to BEL treatment was defined by the requirement of additional therapy or dose increment of ongoing treatment for cGvHD, intolerance to BEL, relapse of the disease for which the alloHCT was performed, and non-relapse mortality [14]. FFS was calculated as a composite endpoint, defined as the time from initiating BEL to adding new GVHD therapy, disease relapse, or death. OS was defined as the time from BEL treatment to death from any cause.

The photographic range of motion (P-ROM) score, which was evaluated as a standard of care, was captured retrospectively and analyzed at 0, 3, and 6 months serially. Significant improvement of the P-ROM score was determined when the score showed improvement by 2 points or higher during BEL therapy.

### Statistical analysis

Patients and disease characteristics were reported using descriptive statistics. The clinical data were locked as of December 2024. Longitudinal changes were analyzed using repeated measures and a general linear model (GLM), evaluating both within-subject and between-subject effects. Proportion comparisons were performed via the Cochran Q test. The incidence of complete discontinuation of steroid was calculated using cumulative incidence method considering competing risks. The Kaplan-Meier estimate was calculated and compared using a log-rank test for FFS and OS, while cumulative incidence was compared using Gray test.

A Cox proportional hazard model was adopted for the 12-month FFS prognostic factor analysis, using hazard ratios (HR) and 95% confidence intervals (95% CI). The following variables were evaluated: conditioning intensity used (reduced versus myeloablative conditioning), prior aGvHD (no versus yes), cGvHD subtype at initial presentation (classical versus overlap syndrome), maximum cGvHD severity (mild/moderate versus severe), fibrotic cGvHD (joint, fascia, lung and MSK involvement; no versus yes), number of organs involved (1 to 3 versus ≥ 4), number of previous GVHD treatment lines (< 3 lines versus ≥ 3 lines), previous RUX use (yes versus no), the reason for failure to last treatment (dependence versus resistance), and treatment modality (BEL monotherapy versus combination of BEL). After the univariate analysis, a multivariate analysis was performed using Cox’s proportional hazard regression model, with a backward stepwise selection procedure applied using a *p* = 0.05 for variable entry and a *p* = 0.10 for variable removal. From those variables identified as significant in the multivariate analysis, a prognostic risk score model was generated by assigning one score to each risk factor, with three groups identified: low risk group without any risk factor, intermediate risk group with one risk factor, and high-risk group with two or more risk factors. Finally, a log-rank test and a Cox proportional hazard regression analysis were performed according to the prognostic risk score model for FFS.

A p-value < 0.05 was considered statistically significant throughout the analysis. The statistical analysis for survival analyses was performed using EZR software v1.64 (Saitama Medical Center, Jichi Medical University, Saitama, Japan) [15].

## Results

### Patient characteristics

A total of 46 patients with cGvHD were treated with BEL, of whom 38 were evaluated for response with a minimum follow-up of 3 months after BEL therapy started. Baseline characteristics are summarized in Table 1. At BEL therapy start, most patients were heavily pre-treated and exhibited severe grade cGvHD (*n* = 28/36, 78%) with a median of 3 affected organs (range, 1–5), and patients had received a median of 4 prior lines of therapy (range, 2–10). The median duration from initial diagnosis of cGvHD to BEL initiation was 23.1 months (range, 1.1–294.5). Corticosteroids were the most concurrently used treatment with BEL (*n* = 31/46, 67%); 18 patients (50%) received BEL in combination with RUX, and two (4%) combined with ECP. Prior treatment failure was noted in 28 patients (61%) with RUX, 10 patients (22%) with IBR, and 11 patients (24%) with extracorporeal photopheresis (ECP). Of the 36 patients with available dosing information for BEL, 32 (89%) had a dose of 200 mg daily, three (8%) received 200 mg twice daily, and one (3%) received 100 mg daily. 

**Table 1:** Summary of demographic and disease characteristics before starting belumosudil therapy and previous treatment of chronic GVHD (*n* = 46)

Category	Groups	No of pts (%)
Patient and disease characteristics		
Sex	Male/Female	28/18 (61/39)
Age at BEL starts	Median (range), year	54.5 (24–74)
	≥ 60 years	17 (47)
Diagnosis at HCT	AML/ALL/T-PLL	18/7/1 (39/16/2)
	CML/MDS/CMML	1/2/1 (2/4/2)
	CLL/NHL/HL	4/2/2 (9/4/4)
	MM/MF/VEXAS	3/4/1 (7/9/2)
Disease status	CR1/≥ CR2 PR/SD	26/6 (57/13) 6/8 (13/17)
Performance status	ECOG 0–1/≥ 2 (*n* = 36)	32/4 (89/11)
	KPS 90–100/< 90	35/11 (76/24)
HCT comorbidity score	Score 0/1/2/3/4/7	16/13/7/6/3/1 (35/28/15/13/7/2)
	HCT-CI score ≥ 3	10 (22)
Transplant characteristics		
Conditioning intensity	Myeloablative conditioning	26 (57)
	Reduced intensity conditioning	20 (43)
HLA match/donor	Mismatched or haploidentical	5 (11)
	Matched related/unrelated	19/22 (41/48)
GvHD prophylaxis	ATG-based PTCy-based PTCy/ATG both Neither ATG nor PTCy	12 (26) 5 (11) 9 (20) 20 (43)
Chronic GvHD characteristics		
Previous history of acute GvHD	Yes	31 (67)
Chronic GvHD subtype	De novo/overlap syndrome	31/15 (67/33)
Chronic GvHD global severity, initial	Mild/moderate/severe	6/20/20 (14/43/43)
No of organ involvement, initial	1/2/3/4/≥ 5	8/14/7/11/6 (17/30/15/24/14)
Chronic GvHD global severity, maximum	Mild/moderate/severe	0/13/33 (0/28/72)
At BEL therapy		
Performance status	ECOG 0/1/2/3	5/27/1/3 (14/75/3/8)
Prior line of cGvHD treatment	1/2/3/4/≥ 5	3/10/14/8/11 (7/22/30/17/24)
Prior treatment to BEL	Ruxolitinib/ECP/ibrutinib	28/11/10 (61/24/22)
Reason for failure to previous treatment	Resistance/intolerance	7/8 (15/18)
	Dependence/others	29/2 (63/4)
Chronic GvHD global severity, at BEL	Moderate/severe	8/28 (22/78)
No. of organ involved, at BEL	1/2/3/4/≥ 5	14/11/10/3/6 (32/25/22/7/14)
Organ involvement (*n* = 36)	Skin/Mouth/Eye/GI Liver/Lung/MSK/Others	22/13/7/6 (61/36/19/17) 15/18/14/1 (42/50/39/3)
Baseline daily prednisone dose (*n* = 26)	Mean ± SE, mg/kg/day	0.16 ± 0.05
Concurrent treatment	Belumosudil monotherapy	26 (57)
	Belumosudil/ruxolitinib	18 (39)
	Belumosudil/ECP	2 (7)
Treatment outcomes with BEL		
Failure		16 (35)
Requiring additional therapy		9 (20)
Non-relapse mortality		1 (2)
Relapse of primary disease		2 (4)
Intolerance		4 (9)
Death		4 (9)
Steroid discontinuation		16/22 (73)
Steroid dose (*n* = 26), mg/kg/day	3 months, mean ± SE	0.10 (0.03)
	6 months, mean ± SE	0.05 (0.01)
	12 months, mean ± SE	0.02 (0.001)
BEL therapy ongoing/discontinued		30/16 (65/35)

*ALL* acute lymphoblastic leukemia, *AML* acute myeloid leukemia, *ATG* anti-thymocyte globulin, *BEL* belumosudil, *CLL* chronic lymphocytic leukemia, *CML* chronic myeloid leukemia, *CMML* chronic myelomonocytic leukemia, *CR* complete remission, *ECOG* Eastern Cooperative Oncology Group, *ECP* extracorporeal photopheresis, *GI* gastrointestinal, *GvHD* graft-versus-host-disease, *HCT* hematopoietic cell transplantation, *HCT-CI HCT* comorbidity index, *HL* Hodgkin’s lymphoma, *HLA* human leukocyte antigen, *NHL* non-Hodgkin’s lymphoma, *MDS* myelodysplastic neoplasm, *MF* myelofibrosis, *MM* multiple myeloma, *MSK* musculoskeletal, *PR* partial response, *PTCy* post-transplant cyclophosphamide, *SD* stable disease, *SE* standard error, *T-PLL* T-cell prolymphocytic leukemia, *VEXAS* vacuoles, E1 enzyme, X-linked, autoinflammatory, somatic syndrome

### Treatment response and long-term outcomes following belumosudil treatment

**Fig. 1:**
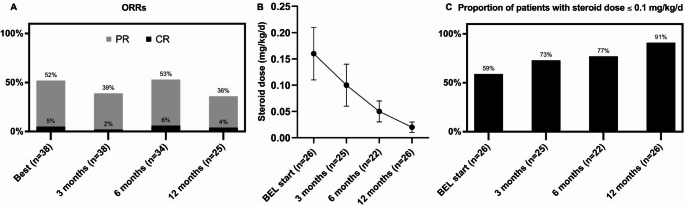
Best overall response rate (ORR) and ORR at 3, 6, and 12 months after belumosudil start (**A**), longitudinal changes in the weight-based corticosteroid dose (**B**), and proportion of steroid ≤ 0.1 mg/kg/d (**C**) after belumosudil start. Legends: 3 M: 3 months; 6 M: 6 months; 12 M: 12 months; BEL: belumosudil; CR: complete response; ORR: overall response rate; PR: partial response

With a median follow-up of 11.4 months (range, 0.2–24) after BEL initiation, the ORR at 6 and 12 months was 53% (*n* = 18/34), and 36% (*n* = 9/25), and the best ORR within 12 months was 52% (*n* = 20/38) (Fig. 1A). At BEL initiation, the mean steroid weight-calculated dose (mg/kg/day) was 0.16 ± 0.05. It progressively decreased when analyzed by the repeated measure using GLM, to 0.10 mg/kg/day ± 0.04 at 3 months, 0.05 ± 0.02 at 6 months, and 0.02 ± 0.01 at 12 months (Fig. 1B) (*p* = 0.02). Moreover, the proportion of patients on a daily steroid dose of ≤ 0.5, 0.2, and 0.1 mg/kg/day was analyzed to assess steroid tapering after BEL start. Although no statistically significant difference was found in the proportion of patients with a daily dose ≤ 0.5 (*p* = 0.112) or ≤ 0.2 mg/kg/day (*p* = 0.072), a statistically significant increase in the proportion of patients ≤ 0.1 mg/kg/day was found (*p* = 0.003; Fig. 1C). Notably, 73% (*n* = 16/22) of the patients discontinued steroids at 12 months. 

**Fig. 2:**
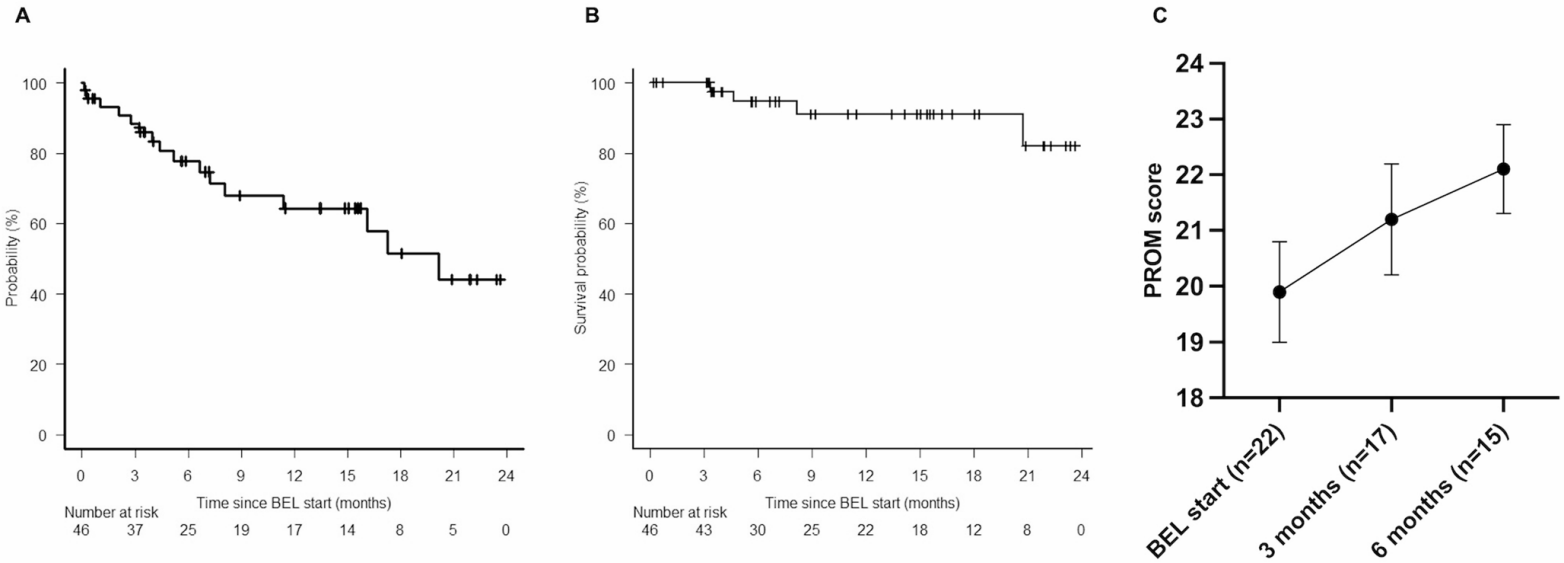
Failure-free (FFS) (**A**) and overall survival (OS) (**B**) at 12 months and longitudinal changes in the photographic range of motion (P-ROM) (**C**). Legends: CI: cumulative incidence; P-ROM: photographic range of motion

Failure to BEL treatment was observed in 16 patients (35%): 9 required additional therapy for cGvHD progression, four due to intolerance (two due to altered liver function tests, one due to muscle weakness, one due to oedema of the limbs), two due to relapse of the primary malignancy, and one died due to progression of pre-existing Mycobacterium avium complex (MAC) lung disease. Considering these events, the FFS was estimated at 64.3% at 12 months (95% CI: 45.9–77.9%) (Fig. 2A); and the OS rate was 91.1% at 12 months (95% CI: 74.6–97.1%) (Fig. 2B).

Out of the 27 patients having available information regarding AEs, none had grade 4 AEs, and only one (4%) had a grade 3 AE due to aspartate (AST) and alanine aminotransferase (ALT) elevation (> 5 times ULN); three (11%) patients had grade 2 AEs: one had bilirubin elevation (> 1.5 times the upper limit of normal [ULN]) and muscle cramps (moderate pain limiting instrumental daily activities), one had muscle weakness of the limbs (symptomatic, evident on physical exam, limiting instrumental daily activities), and one had oedema of the limbs (apparent deviation from normal anatomic contour; limiting instrumental daily activities).

### Photographic range of motion (P-ROM) measurements

The P-ROM measurements at baseline were available in 24 patients. The P-ROM was gradually improving over time and significantly when analyzed by repeated measures using GLM up to 6 months after BEL start (*p* = 0.007): 19.9 ± 0.9 at BEL start, 21.2 ± 1.0 at 3 months and 22.1 ± 0.8 at 6 months (Fig. 2C).

### Impact of combination therapy of belumosudil with ruxolitinib or extracorporeal photopheresis

Because the mechanism of action of BEL is different from RUX or from ECP, many practitioners attempted to utilize BEL in combination with either RUX or ECP in the treatment of cGvHD. Thus, we analyzed the treatment outcomes of BEL/combination and compared the outcomes between patients treated with BEL/combination therapy (*n* = 20) vs. those treated with BEL monotherapy (*n* = 26). For the 20 patients with available data on their RUX dose, 15 (75%) received 10 mg twice daily, three (15%), 5 mg twice daily, and one each (5%) received 15 mg twice daily or 5 mg daily. For BEL, of 36 patients with available data, 32 (89%) received 200 mg daily, three (8%) 200 mg twice daily, and one (3%) 100 mg daily.

When compared, the characteristics of the patients treated with BEL/combination therapy to those with BEL alone, BEL/combination group showed a higher proportion of patients having four or more organs involved with cGvHD (55% vs. 23%, *p* = 0.035) and a higher number of patients who failed previous treatment with RUX (85% vs. 42%, *p* = 0.006) (Table 2). Interestingly, in this context, no difference was found between the two groups regarding dosage (86% with 200 mg daily vs. 91%, *p* = 0.441), ORR (41% vs. 62%, *p* = 0.328), BEL failure rate (40% vs. 31%, *p* = 0.548), BEL intolerance rate (5% vs. 11%, *p* = 0.622), or steroid discontinuation rate (43% vs. 57%, *p* = 1.000).

**Table 2:** Comparison between belumosudil combination therapy and belumosudil monotherapy

Category	Groups	BEL combination (*n* = 20)	BEL monotherapy (*n* = 26)	*p*-value
Follow-up duration, days, median (range)		431 (104–713)	277 (5–720)	0.073
Patients’ characteristics				
Sex	Male/Female	13/7 (65/35)	15/11(42/58)	0.763
Age at BEL starts	Median (range), year	58 (24–74)	54 (24–73)	0.947
	≥ 60 years	9 (45)	9 (35)	0.550
Performance status	ECOG 0–1/≥ 2	17/1 (94/6)	15/3 (83/17)	0.603
HCT-CI	HCT-CI score ≥ 3	5 (25)	5 (19)	0.726
Chronic GvHD characteristics at belumosudil				
Previous acute GvHD	Yes	13 (72)	16 (62)	0.365
HCT to cGvHD, initial	Days, median (range)	159 (50–750)	187 (105–1072)	0.513
HCT to belumosudil	Days, median (range)	1099 (384–4486)	889 (256–9907)	0.236
cGvHD to belumosudil	Days, median (range)	792 (217–4214)	654 (33–8835)	0.298
Chronic GvHD subtype	De novo/overlap syndrome	10/10 (50/50)	21/5 (81/19)	0.055
cGvHD maximum severity	Moderate/severe	5/15 (25/75)	8/18 (31/69)	0.749
No of organ involvement	≥ 4	11 (55)	6 (23)	**0.035**
Prior treatment to BEL	1–2 line(s)/≥ 3 lines	7/13 (35/65)	6/20 (23/77)	0.511
	Ruxolitinib	17 (85)	11 (42)	**0.006**
	ECP	6 (30)	5 (19)	0.494
	Ibrutinib	4 (20)	6 (23)	1.000
Reason for previous treatment failure	Resistance or intolerance	7 (35)	8 (31)	1.000
	Dependence	13 (65)	18 (69)	-
Organ involvement (*n* = 36)	Skin/Mouth/Eye/GI	16/15/8/6 (89/83/44/33)	14/9/6/2 (78/50/33/11)	NS
	Liver/Lung/MSK/Others	13/9/7/3 (72/50/39/17)	9/10/4/0 (50/56/22/0)	NS
Fibrotic GvHD (*n* = 36)		11 (61)	13 (72)	0.725
Treatment outcomes with BEL				
Best ORR		7/17 (41)	13/21 (62)	0.328
ORR at 3 months		6/17 (35)	9/21 (43)	0.744
ORR at 6 months		6/15 (40)	12/19 (63)	0.300
ORR at 12 months		3/12 (25)	6/13 (46)	0.411
Failure with BEL therapy		8 (40)	8 (31)	0.548
Requiring additional therapy		5 (25)	4 (15)	0.472
Non-relapse mortality		1 (5)	0 (0)	0.432
Relapse of primary disease		1 (5)	1 (4)	1.000
Intolerance		1 (5)	3 (11)	0.622
Death		2 (11)	2 (11)	1.000
Steroid dose, mg/kg/day	Baseline, mean ± SE	0.14 ± 0.22	0.18 ± 0.31	0.740
	3 months, mean ± SE	0.07 ± 0.11	0.13 ± 0.23	0.441
	6 months, mean ± SE	0.06 ± 0.07	0.05 ± 0.09	0.749
	12 months, mean ± SE	0.03 ± 0.06	0.01 ± 0.03	0.168
BEL therapy ongoing/discontinued		11/9 (55/45)	19/7 (73/27)	0.229

*BEL* belumosudil, *ECOG* Eastern Cooperative Oncology Group, *ECP* extracorporeal photopheresis, *GI* gastrointestinal, *GvHD* graft-versus-host-disease, *HCT* hematopoietic cell transplantation, *HCT-CI* HCT comorbidity index, *MSK* musculoskeletal, *NS* non-significant, *ORR* overall response rate, *RUX* ruxolitinib, *SE* standard error

Accordingly, no difference was noted for FFS (62.9% vs. 65.6%, *p* = 0.991) or OS (92.9% vs. 89.7%, *p* = 0.847) at 12 months between the two groups. After adjusting for confounding factors (i.e. moderate-severe grade cGvHD, ≥ 4 organ involvement by cGvHD, and previous RUX therapy), the FFS rate at 12 months was 39.6% for the BEL/combination group and 61.3% for the BEL monotherapy group. When looking strictly at the groups treated with BEL/RUX combination therapy and BEL monotherapy, a higher proportion of patients had four or more organs involved with cGvHD (61.1% vs. 23.1%, *p* = 0.015), and previous RUX treatment in the combination group (89% vs. 42%, *p* = 0.002), while no differences in the treatment outcomes were found (data not shown).

### Risk factor analysis for failure-free survival following belumosudil treatment

When analyzing the risk factors for BEL failure, the univariate analysis identified the following risk associated with FFS following BEL treatment: (1) cGvHD involvement in more than four organs (HR 10.07, [1.69 − 60.08], *p* = 0.011) was linked to worse FFS; (2) previous episode of aGvHD (HR 7.58, [0.87–65.57], *p* = 0.066) suggested a trend towards worse FFS; (3) the presence of fibrotic component of cGvHD (HR 0.13, [0.02–1.00], *p* = 0.051) showed a trend towards a better FFS (Table 3). In the multivariate analysis, only having more than four organs affected by cGvHD (HR 3.42, [1.14–10.20], *p* = 0.028) continued to impact FFS negatively, while previous episode of aGvHD (HR 6.77, [0.88–52.33], *p* = 0.06) was associated to a trend towards worse FFS.

**Table 3:** Univariate and multivariate analysis for failure-free survival following belumosudil therapy

Category	Risk group	*n*	12-mo FFS (%)	Univariate analysis		Multivariate analysis	
				HR (95% CI)	*p*-value	HR (95% CI)	*p*-value
Conditioning intensity	RIC	20	73.8	-	-	-	-
	MAC	26	58.8	0.55 (0.11–2.77)	0.471	-	NS
Previous aGvHD	No	15	92.3	-	-	-	-
	Yes	31	53.1	7.58 (0.87–65.57)	0.066	6.77 (0.88–52.33)	0.066
cGvHD subtype	Classic	31	57.7	-	-	-	-
	Overlap	15	76.0	0.28 (0.04–1.77)	0.175	-	NS
Maximum cGvHD severity	Mild/moderate	13	88.9	-	-	-	-
	Severe	33	55.7	3.13 (0.48–20.27)	0.116	-	NS
Fibrotic cGvHD	No	12	40.2	-	-	-	-
	Yes	24	73.7	0.13 (0.02–1.00)	0.051	-	NS
No. of organs involved	1–3	29	78.8	-	-	-	-
	≥ 4	17	48.5	**10.07 (1.69–60.08)**	**0.011**	**3.42 (1.14–10.20)**	**0.028**
cGvHD treatment lines	1–2	13	63.3	-	-	-	-
	≥ 3	33	65.5	0.93 (0.14–6.15)	0.259	-	NS
Previous ruxolitinib use	No	18	63.8	-	-	-	-
	Yes	28	64.8	0.36 (0.06–2.12)	0.259	-	NS
Last treatment failure	Dependence	31	67.8	-	-	-	-
	Resistance	15	42.3	0.42 (0.05–3.56)	0.431	-	NS
Belumosudil monotherapy	Yes	26	65.6	-	-	-	-
	No	20	62.9	1.23 (0.27–5.55)	0.783	-	NS

95% *CI* 95% confidence interval, *aGvHD* acute graft-versus-host-disease, *cGvHD* chronic graft-versus-host-disease, *ECOG* Eastern Cooperative Oncology Group, *GI* gastrointestinal, *GvHD* graft-versus-host-disease, *FFS* failure-free survival, *HCT* hematopoietic cell transplantation, *HCT-CI* HCT comorbidity index, *HR* hazard ratio, *MAC* myeloablative conditioning, *NS* non-significant, *RIC* reduced-intensity conditioning

**Fig. 3:**
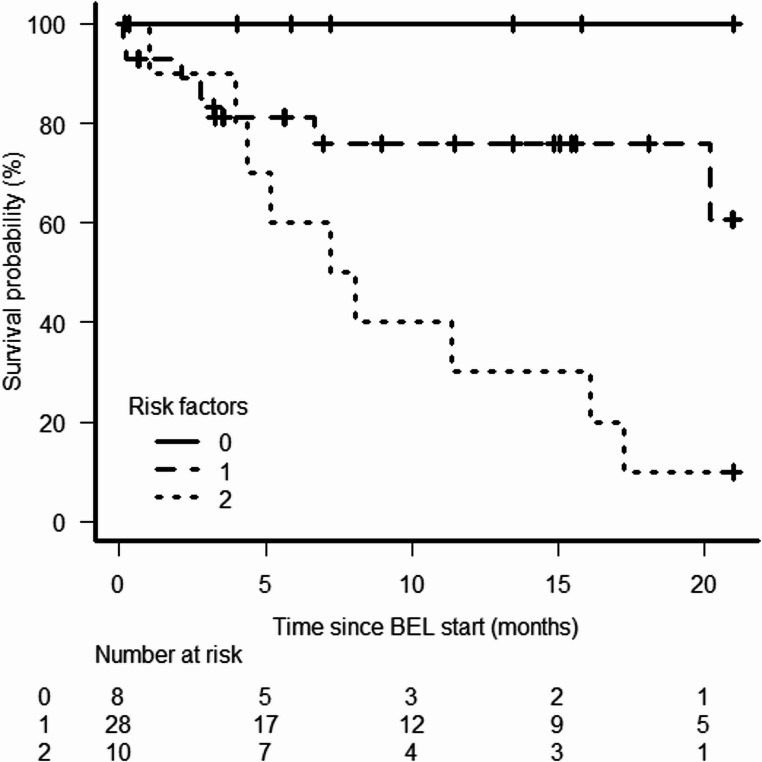
Risk score analysis incorporating previous acute graft-versus-host-disease (GvHD) and organ involvement by chronic GvHD after belumosudil start stratified according to their risk for failure-free survival (**A**). Legends: BEL: belumosudil

We developed a prognostic risk score incorporating the risk factors identified above: (1) more than four organ involvement by cGvHD and (2) previous episode of aGvHD. The proposed risk score could stratify patients with respect to FFS: 100% in low risk group (patients without any risk factors, *n* = 8), 75.8% (95% CI 53.3–88.6%) in intermediate risk group (patients with one risk factor, *n* = 28) and 30% (95% CI 7.1–57.8%) in high risk patients (patients with the two risk factors, *n* = 10) (HR 3.91, 95% CI 1.58–9.67, *p* = 0.006) (Fig. 3).

## Discussion

This multicentre retrospective study reports that BEL: (1) demonstrates sustained efficacy, achieving an ORR of 52% and a FFS rate of 64% at 12 months; (2) is a well tolerated treatment option for heavily pre-treated patients, with only one grade ≥ 3 toxicity reported (hepatic, in the BEL monotherapy group); (3) risk factors for FFS on BEL was identified: 4 or more organ involved by cGvHD, and previous episode of aGvHD; (4) BEL combination therapy is feasible.

Our results are consistent with the findings from the pivotal ROCKstar trial (KD025-213), where BEL achieved an ORR of 74% and an FFS rate of 56% at 12 months in patients with SR-cGvHD [3], despite our study comprising patients with multiple failures to the previous lines of cGvHD treatment. Similarly, our results align with several recent RWD studies showing comparable ORR and FFS rates at 12 months (Table 4). Compared to other cGvHD therapies such as RUX or ibrutinib, associated with a higher rate of grade ≥ 3 cytopenias (35%) in the REACH3 trial [20], and to increased bleeding and infections [21], BEL was well tolerated in the current patient population, with failure reported in only 16 patients (35%), four due to intolerance, and only one grade ≥ 3 (4%) hepatic toxicity during follow-up, highlighting the manageable toxicity profile of BEL. Moreover, it may offer a safer long-term option for patients with cGvHD, especially those who have experienced hematologic toxicity with prior therapies, as well as facilitate prolonged treatment duration, potentially contributing to this study’s sustained symptom improvement and durable responses. Given the chronic nature of cGvHD and the need for long-term therapy, maintaining a balance between efficacy and safety is critical for optimizing patient outcomes.

**Table 4:** Real-world data of belumosudil in chronic graft-versus-host disease

Country	Present study	US/Ohio [16]	US/MSK [17]	US/COH [18]	Germany [10]	France [11]	Spain	Israel [19]
Year	2025	2024	2024	2025	2025	2025	2025	2025
No of pts	46	20	66	45	33	68	63	45
ORR, 6mo	53%	-	59%		-	46%	-	-
CR, 6mo	6%	-	5%		-	10%	-	-
Best ORR	53%	55%	64%	47%	42%	57%	62%	55.6%
Best CR	5%	5%	10%	7%	-	15%	5%	6.7%
FFS, 12 months	64%	-	72%	46%	64%	80%	-	72%
OS, 12 months	91%	-	92%	84%	88%	96%	-	92%
Organ	-	-	-	-	GI 67%	Liver/mouth 70%	Mouth 75%	-
Lung	-	-	-	-	23%	-	30%	44.4%
MSK	-	-	-	-	25%	-	53%	61.1%
Toxicities	Liver G3 (1)	-	No ≥ Gr3	-	-	-	Liver	-
Infections	-	-	-	-	21%	-	27%	17.8%

*ASTCT* American Society for Transplantation and Cellular Therapy, *COH* City of Hope, *MSK* Memorial Sloan Kettering, *US* United States

Among patients who failed prior RUX therapy, optimal management using molecules acting on different pathways is an emerging issue, as it is still unclear how to best transition to BEL treatment after RUX failure: to switch to BEL monotherapy while stopping RUX, or to add BEL to RUX. In our study with 16 pts (35%) with previous RUX failure (compared to 63% in the ROCKstar study), BEL in combination with RUX was feasible and effective. The higher FFS in our study may reflect a synergistic activity: emerging data suggest that BEL combined with RUX or ECP may produce therapeutic synergism by simultaneously targeting distinct mechanisms of cGvHD pathogenesis [9, 13, 22], utilizing the antifibrotic property of BEL and the strong anti-inflammatory activity of RUX without increasing toxicity. However, it has to be carefully evaluated prospectively with a pre-determined treatment plan, particularly with respect to toxicity and tolerability from combination therapy of BEL and RUX.

Sustained improvement was observed beyond six months in our study, with a mean improvement of 2 points in the P-ROM scores (from 19.9 to 22.1) from the start of BEL to 6 months, highlighting the potential for BEL to deliver a sustained reduction in cGvHD-related symptoms burden. Similar results with BEL’s impact on P-ROM are lacking. Regarding other agents, a single-center retrospective study that included patients with refractory sclerodermatous cGVHD showed an improvement in P-ROM after RUX therapy [23]. Equally, a prospective, multicenter study supported the use of both the NIH joint/fascia scale and the P-ROM scale for joint and fascia manifestations [24].

We identified two risk factors for BEL failure: a history of aGvHD, and four or more organ involvement by cGvHD, which stratified patients with a worse FFS. Previous attempts for risk scores that adequately predict FFS have not shown promising results [25]. Only a retrospective study reported that a severe NIH global score (which considers the number and severity of organs affected) at second-line treatment was associated with worse FFS, but found no difference in outcomes in patients with four or more sites involved [26]. Although they are well-known risk factors for higher-risk GvHD, their presence in a heavily pre-treated population may help guide decisions regarding BEL combination therapy.

Our study has several limitations. The inherent restraints associated with its retrospective nature are mainly bound to the accuracy of the medical record, failing to assess toxicity data beyond laboratory values, and the risk of response rate overestimation. Yet, our analysis overcomes limitations by the objectivity of the FFS components, such as death, relapse, or the need for a subsequent line of therapy, as well as its multicenter nature, limiting bias associated with single-center patterns and enhancing the generalizability of our findings.

In summary, BEL offers sustained efficacy resulting in an acceptable ORR and a high FFS, along with clinically meaningful symptom improvements, corticosteroid withdrawal, and a favorable safety profile in a heavily pre-treated cGvHD population, showing promise as a salvage therapy for refractory cGvHD after multiple lines of treatment. A simple risk-score assessment based on prior aGvHD and the extent of organ involvement predicted FFS associated with BEL treatment. Prospective trials looking at the optimal dosing, timing, and bridging with extended follow-up duration are recommended to confirm these results and better define long-term outcomes. Future investigations should also explore different combination approaches and identify biomarkers to optimize patient selection and enhance therapeutic efficacy.

## Data Availability

The data supporting this study are not publicly available due to Canadian privacy law, but may be available upon reasonable request to the corresponding author and subject to institutional approval.

## Declarations

### Data statement

The data supporting this study are not publicly available due to Canadian privacy law, but may be available upon reasonable request to the corresponding author and subject to institutional approval.
